# Comparative Evaluation of Freezing Protocols and Cryopreservation Media for the Preservation of Alginate Encapsulated HepaRG Cells

**DOI:** 10.3390/cells15191783

**Published:** 2026-09-30

**Authors:** Carla Meschini, Andrea Bartolucci, Ulysse Pereira

**Affiliations:** 1 UMR CNRS, BMBI, Génie Biologique, Université de Technologie de Compiègne, F-60203 Compiègne, France; ulysse.pereira@utc.fr; 2 University Campus Bio-Medico di Roma, 00128 Rome, Italy; andrea.bartolucci@santannapisa.it

**Keywords:** hepatocytes, HepaRG cells, alginate, cryopreservation, controlled-rate-freezing, cryopreservation solution

## Abstract

Reliable storage and distribution are important for developing ready-to-use encapsulated cell products for transplantation and bioartificial liver applications. This study evaluated the cryopreservation of alginate-encapsulated HepaRG cells using a programmable controlled-rate freezing (CRF) protocol at −0.3 °C/min or conventional passive cooling at approximately −1 °C/min. Five media were tested: four commercial xeno-free formulations, including two DMSO-containing and two DMSO-free media, and a conventional formulation containing 90% fetal bovine serum and 10% DMSO. Following thawing and a 3-day recovery period, metabolic activity, albumin secretion, ammonia and lactate clearance, and phase I and phase II biotransformation activities were assessed. Cryopreservation reduced metabolic activity relative to non-frozen controls, but key hepatic functions remained detectable. The tested CRF protocol generally yielded equal or higher functional values than conventional freezing. Medium performance varied according to the endpoint assessed. Within CRF, CryoStor CS10 produced relatively high mean PrestoBlue and albumin secretion values, whereas Cellvation produced the highest mean CYP1A1/1A2 activity. These product-specific observations do not establish a general effect of DMSO. The findings support the short-term feasibility of the tested CRF protocol for this encapsulated HepaRG model but not broader clinical or drug-testing applications.

## 1. Introduction

Alginate encapsulation has primarily been developed for cell transplantation and bioartificial liver applications, where the hydrogel can provide physical immunoprotection and facilitate handling and storage [1,2,3]. The increasing demand for advanced in vitro models, driven by the need for improved physiological relevance and adherence to the 3Rs (replacement, reduction, and refinement of animal experimentation), has accelerated the development of three-dimensional (3D) cell-culture systems in fundamental research, drug development, and regenerative medicine. Compared with conventional two-dimensional cultures, 3D cellular models better reproduce tissue architecture, cell–cell interactions, and physiological functions, thereby improving the predictive value of in vitro studies [4,5,6,7].

Hepatic cells express differences in gene and protein expression depending on whether they are cultured in 2D or 3D environments. Indeed, the mechanical properties of the microenvironment and the signaling crosstalk with the extracellular matrix (ECM) impact the cellular differentiation [8]. The aim of 3D cell culture is to provide a microenvironment that more closely resembles in vivo conditions, thereby creating more representative models for preclinical studies, drug screening, and disease modeling [9,10].

Despite these advantages, the widespread implementation of encapsulated cellular systems remains limited by their high production costs, labor-intensive maintenance, and relatively short lifespan. Repeated production batches may generate significant inter-batch variability due to differences in cell quality, differentiation status, culture conditions, or construct maturation [11,12]. Such variability can compromise experimental reproducibility and hinder large-scale implementation of these models in research and industrial settings. Consequently, the development of robust cryopreservation strategies is essential to enable the long-term storage, distribution, and standardization of encapsulated systems. Efficient cryopreservation would facilitate the establishment of ready-to-use cell banks, reduce production costs, improve batch consistency, and increase accessibility of complex cellular systems.

However, the cryopreservation of complex alginate cell constructs remains particularly challenging because of several drawbacks such as: ice crystal formation, osmotic stress, cryoprotectant toxicity and freeze-induced mechanical damage [13,14,15,16]. This phenomenon happens during freezing and can compromise the structural integrity and viability of these complex constructs [17]. The stresses caused can thus impair cellular viability and metabolic activity, ultimately limiting the functional relevance and immediate usability of tridimensional models following thawing.

Different strategies have been investigated to improve post-thaw recovery and preserve liver-specific functions. In cell culture, encapsulation of cells inside a biomaterial such as alginate has been used for a wide period of time because it presents several advantages in vitro [18,19] and recently in vivo [3,20]. Alginate-based microencapsulation has emerged as a promising approach for providing a three-dimensional microenvironment favorable for the maintenance of hepatic phenotype [21]. More recently, studies showed that hydrogels may also confer physical protection against freezing-induced damage [22]. Encapsulation within an alginate matrix therefore represents a practical strategy for the cryopreservation of 3D structures, providing protection against freezing damage and structural support and facilitating handling and storage. In encapsulated systems, where alginate influences heat and mass transfer, optimizing the cooling rate is particularly critical [23,24]. The cooling rate was selected based on studies showing that slower freezing improves cell survival and function compared to the conventional −1 °C/min [25,26], where cooling rates between 0.25 and 0.5 °C/min have been explored, supporting the use of a −0.3 °C/min rate.

However, the protective effect of the biomaterial alone is not sufficient to ensure optimal post-thaw recovery. The thermal history experienced by the construct during freezing is another critical determinant of cryopreservation success, as cooling kinetics directly influence water transport, osmotic stress, and intracellular ice formation. Consequently, the choice of freezing strategy must be considered together with the cryoprotective formulation when developing an optimized cryopreservation protocol for encapsulated 3D cells. Different freezing strategies can be used for cell cryopreservation, ranging from conventional freezing methods to more controlled approaches such as controlled-rate freezing (CRF), each influencing intracellular ice formation, osmotic stress and post-thaw cell recovery. In this context, CRF offers several advantages over conventional freezing, including better regulation of ice formation, reduced osmotic stress, and improved reproducibility of the freezing process, thereby supporting the maintenance of cellular morphology and functional activity after thawing [27,28]. Additionally, the use of a programmable controlled-rate freezer allows the simultaneous processing of a larger number of cryovials and greater sample volumes, thereby enhancing reproducibility and scalability for downstream applications.

However, although these parameters improve cryopreservation outcomes, the choice of freezing medium and cryoprotective agents remains a critical determinant of successful preservation strategies. Conventional cryopreservation formulations are still largely based on fetal bovine serum (FBS) and dimethyl sulfoxide (DMSO), both of which present important limitations for reproducibility and translational applications. FBS is commonly used as a supplement in freezing media due to its protective properties; however, its undefined composition and batch-to-batch variability raise significant concern [29]. In addition, its xenogeneic origin poses risks of pathogen transmission and immune reactions, making it unsuitable for clinical translation [30]. These limitations are particularly relevant for hepatic models, where functional readouts are highly sensitive to microenvironmental variations [31]. DMSO remains the standard permeating cryoprotectant owing to its efficacy in limiting intracellular ice formation. Nevertheless, its use is associated with well-documented cytotoxic effects (neurotoxicity [32] and functional effects [33,34,35]), including alterations in cell viability and modulation of hepatic gene expression programs. In hepatic systems, residual DMSO after thawing may also confound metabolic and functional assessments [36]. Collectively, these limitations highlight the need for xeno-free and reduced-DMSO or DMSO-free cryopreservation formulations, particularly for the preservation of hepatic aggregates intended for functional and toxicological studies.

This study compared post-thaw outcomes across freezing protocols and cryopreservation media for alginate-encapsulated HepaRG cells. Two freezing approaches were compared: programmable controlled-rate freezing (CRF; −0.3 °C/min, including a 15 min hold at −10 °C) and conventional passive freezing (approximately −1 °C/min). These freezing protocols were evaluated in combination with five cryopreservation media: four commercially available xeno-free formulations, including two DMSO-containing and two DMSO-free media, and a conventional formulation composed of 90% fetal bovine serum (FBS) and 10% DMSO. Following cryopreservation, the constructs were thawed and allowed to recover for 3 days before metabolic activity and liver-specific functions were assessed. The selection of this recovery time point was based on previous studies demonstrating that hepatic models require up to 72 h after thawing [37,38,39]. Morphology, viability, and function were compared between parallel unfrozen controls and post-thaw samples; the same beads were not measured before and after cryopreservation.

## 2. Materials and Methods

### 2.1. Cell Culture

Human hepatocyte lineage HepaRG^®^ cells (Biopredic International, Rennes, France) were cultured according to the manufacturer’s instructions. Cells were maintained in William’s E phenol red-free medium (Gibco) supplemented with HepaRG growth additive (ADD710C, Biopredic International, Rennes, France). The cells were seeded at an initial density of 2.5 × 10^4^ cells/cm^2^ and maintained at 37 °C in a humidified atmosphere containing 5% CO_2_. The medium was renewed three times per week until full confluency and differentiation at 14 days of culture. For further expansion or experimental use, HepaRG cells were detached enzymatically using 0.25% trypsin-ethylenediaminetetra acetic acid (EDTA) (Gibco, Thermo Fisher Scientific, Grand Island, New York, NY, USA).

### 2.2. Alginate Microencapsulation

Cells were enzymatically detached from the Petri dishes and resuspended in 1 mL of 1.5% (*w*/*v*) alginate solution (Manucol LKK (FMC BioPolymer, Billingstad, Asker, Norway) (Guluronic/Mannuronic ratio 30:70), sterilized with successive filtrations with 0.8, 0.45, and 0.22 µm pore size membrane filters (Merck Millipore, Burlington, MA, USA) at a density of 10 × 10^6^ cells per mL. The suspension was loaded into a 5 mL syringe connected to the air-jet encapsulation setup linked to a 24 G nozzle. The air pressure was adjusted to 3.5 units, and the syringe pump was set to a flow rate of 9.5 mL/min. Uniform droplets were generated by coaxial air flow, enabling the needle to vibrate inside a nozzle. Beads were collected directly in a 154 mM CaCl_2_ gelling bath. Beads were allowed to polymerize for 15 min at 4 °C, then washed with sterile culture medium to remove excess calcium ions. Encapsulated cells were maintained in HepaRG complete medium in a shaking plate (75 RPM), with medium replacement three times per week. Cultures were maintained for up to 13 days prior to analysis. One batch of encapsulation was composed of serial encapsulations of 750,000 cells/750 µL of sterile sodium alginate 1.5% (*w*/*v*).

**Figure 1:**
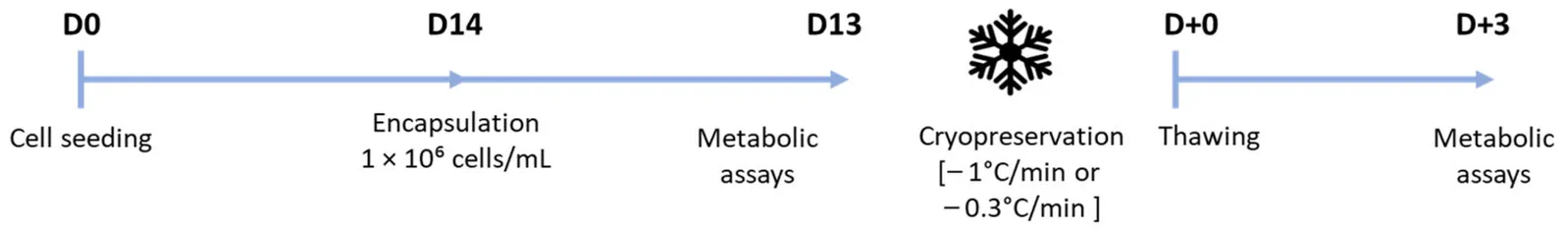
Schematic timeline summarizing the experimental workflow of the study.

The experimental timeline is illustrated in Figure 1. Cells were first cultured in alginate beads for 13 days and subsequently cryopreserved. After 7 days of cryopreservation, the cells were thawed and allowed to undergo a 3-day recovery period. Non-destructive metabolic assays were then performed to assess their functional recovery after cryopreservation.

These analyses aimed to evaluate the metabolic activity and viability of the cells after thawing, allowing comparison between the different batches cryopreserved with a specific CPA and providing insight into the impact of each cryopreservation medium on hepatocyte functionality. Following this pre-culture period, cryopreservation was performed using either the controlled-rate freezing protocol (−3 °C/min, 15 min at −10 °C and then −3 °C/min) or conventional passive cooling (−1 °C/min). After thawing, encapsulated hepatocytes were recovered and cultured for an additional 3 days to assess post-thaw recovery, viability, and functional activity [40]. Morphology, viability, and function were compared using samples originating from the same large encapsulation batch, which was divided into separate lots, assigned to the pre-cryopreservation control and the different cryopreservation conditions.

### 2.3. Cryopreservation and Experimental Design

To evaluate the influence of cryoprotective composition and freezing conditions on the post-thaw recovery of alginate-encapsulated HepaRG, four commercial cryopreservation agents were compared with an in-house-prepared cryopreservation medium containing 90% animal serum (FBS) and 10% dimethyl sulfoxide (Table 1). Every commercial cryoprotective medium used was serum-free and xeno-free, but half of them were DMSO-free and the other half contained 10% DMSO. In total, five different cryopreservation media were compared during our experiments.

To minimize inter-batch variability, the experiments were performed using several independently prepared batches of alginate-encapsulated HepaRG cells. For each independent experiment, one large encapsulation batch was produced under the same culture and encapsulation conditions and subsequently divided into matched aliquots assigned to the unfrozen control and the different combinations of cryopreservation media and freezing protocols. Thus, all conditions compared within a given experiment originated from the same HepaRG culture and encapsulation batch. This paired experimental design minimized the influence of culture and encapsulation-related variability, allowing differences between matched conditions to be primarily associated with the cryopreservation procedures. Each independently prepared batch was considered one biological replicate, whereas measurements performed on aliquots derived from the same batch were considered technical replicates and were averaged before statistical analysis.

Following addition of the cryoprotective agent, samples were incubated for 15 min at 4 °C to reduce thermal shock, allow CPA diffusion, and ensure a homogeneous initial temperature. After equilibration, samples were transferred to Nalgene^®^ cryogenic vials (Nalgene, Waltham, MA, USA) and subjected to one of two complete freezing protocols. Because the protocols differed in more than nominal cooling rate, they were compared at the protocol level.

For conventional freezing, cryovials were placed in CoolCell™ LX freezing containers (Corning Inc., NY, USA) and transferred directly to a −80 °C freezer. This passive cooling system provides an approximate cooling rate of −1 °C/min until the samples reach −80 °C. After 24 h, cryovials were transferred into liquid nitrogen tanks for long-term storage at −196 °C.

For CRF, samples were frozen using a CryoMed™ programmable controlled-rate freezer (Thermo Fisher Scientific, Waltham, MA, USA). The controlled cryopreservation followed a program composed of [−0.3 °C/min until −10 °C; temperature hold for 10 min to ensure ice nucleation; −0.3 °C/min until −80 °C]. At the end of each freezing protocol, samples were immediately transferred to liquid nitrogen storage (−196 °C) for 7 days until further analysis.

Thawing of the samples was realized as follows: 1 min in a 37 °C waterbath with gentle shaking until dissolution of 90% of ice in the cryotube and transfer of the cells into 10 mL of warm complete medium. Then, the cells are washed and placed into culture for 3 days until the metabolic assays are performed.

### 2.4. Bead Morphology and Viability Assay

The morphology of the beads was monitored using a phase-contrast microscope until three days after thawing. Cell viability was assessed using a Live/Dead^®^ fluorescence staining assay (cat. no. L3224, lot no. 2942297; Invitrogen, Thermo Fisher Scientific, Eugene, OR, USA). A negative control for cell viability was produced by incubating a sample with 70% ethanol during the 1 h prior to perform the Live/Dead^®^ Assay. Briefly, cells (including the control) were incubated for 45 min at 37 °C under agitation (70 RPM) in a staining solution containing 10 µM calcein-AM, 20 µM ethidium homodimer-1 (EthD-1) and 6 µM Hoechst 33342 (20 mM; cat. no. 62249; Thermo Fisher Scientific GmbH, Dreieich, Germany) prepared in William’s E medium. After incubation, cells were washed with PBS, and images were acquired as Z-stacks for each sample using confocal fluorescence microscopy (ZEISS LSM 980 Airyscan; Carl Zeiss Microscopy GmbH, Jena, Germany). For visualization, the images are presented as maximum intensity projections (MIPs), which represent a two-dimensional reconstruction obtained by projecting all focal planes to a single image. Viable cells appeared green (calcein), dead cells red (EthD-1), and nuclei blue (Hoechst). Image analysis was performed using Zeiss Zen v3.9 (Zen lite) software.

### 2.5. Metabolic Activity Measurement by Presto Blue HS Assay

Metabolic activity was measured using the non-destructive PrestoBlue™ HS Cell Viability Reagent (cat. no. P50201; Invitrogen, Thermo Fisher Scientific, Waltham, MA, USA). This reagent is based on the redox dye resazurin, which is cell-permeable and non-toxic. In viable cells, mitochondrial enzymes convert a non-fluorescent substrate: resazurin (blue, non-fluorescent) converts a fluorescent product (resorufin) by accepting electrons from cellular metabolic processes. Briefly, cells were washed twice with PBS, then incubated in a PrestoBlue HS solution prepared in serum-free William’s E medium. Then, 1 mL of the solution was collected before and after 15 min of incubation at 37 °C under agitation. Fluorescence was measured using a microplate reader (SpectraFluor Plus, Tecan Group AG, Männedorf, Switzerland) (λ_ex_ = 535 nm, λ_em_ = 595 nm). A standard curve of known resorufin concentration was used to quantify metabolic activity.

### 2.6. Assessment of Hepatic Functions in Vitro

#### 2.6.1. Albumin Secretion Assay (ELISA)

Albumin secretion was quantified using a human albumin ELISA kit (cat. no. E88-129 Bethyl Laboratories, Montgomery, TX, USA). Culture supernatants were collected at baseline (T0) and after 6 h of incubation to measure secretion per hour, calculated from the difference between T6h and T0 values. Supernatants were centrifuged and stored at −80 °C until analysis. Data are reported as mean ± SD from three independent biological experiments and four for the Presto Blue HS assay.

#### 2.6.2. Xenobiotic Activity—Phase I Metabolism

Cytochrome P450 (CYP)-mediated xenobiotic biotransformation activity (Phase I) was assessed by measuring CYP1A1/2 and CYP3A4 enzyme activities using the ethoxyresorufin-O-deethylase (EROD) assay. Briefly, 7-ethoxyresorufin, a non-fluorescent substrate, is O-dealkylated by CYP enzymes to generate resorufin, a red fluorescent product. Cells were incubated with 10 µM of the respective substrate for 2 h at 37 °C on day 3 post cryopreservation. Culture supernatants were collected at baseline (T0) and after 2 h of incubation to assess cellular metabolic activity. Metabolic changes were calculated from the difference between T2h and T0 values. To prevent interference between Phase I and Phase II enzymes, culture media were supplemented with 3 mM salicylamide and 40 µM dicumarol (cat. no. M1390; Sigma-Aldrich, Steinheim, Germany). Following incubation, supernatants were collected and stored at −20 °C until analysis. Resorufin fluorescence was measured using a SpectraFluor Plus spectrometer (TECAN, Männedorf, Switzerland) at excitation and emission wavelengths of 535 nm and 595 nm, respectively. A standard curve was generated using a 10 µM resorufin solution to determine the metabolite concentrations in the samples (as first described by M. D. Burke & R. T. Mayern, 1974) [41].

#### 2.6.3. Xenobiotic Activity—Phase II Metabolism

UGT1A1 (uridine diphosphate glucuronosyltransferase 1A1) activity was quantified following a protocol adapted from Leite et al. (2012) [42]. Briefly, cells were incubated for 1 h at 37 °C in the presence of 100 µM 4-methylumbelliferone (4-MU; cat. no. M1381; Sigma-Aldrich, St. Louis, MO, USA). After incubation, supernatants were collected and stored at −20 °C until analysis. 4-MU, a fluorescent substrate, is converted by UGT1A1 into a non-fluorescent glucuronide conjugate. The residual fluorescence corresponding to non-conjugated 4-MU was measured using a fluorescence spectrometer (TECAN, Männedorf, Switzerland) at excitation and emission wavelengths of 320 nm and 450 nm, respectively. UGT1A1 activity was determined by calculating the amount of glucuronidated 4-MU, derived from the decrease in initial substrate concentration explained in [43]. Culture supernatants were collected at baseline (T0) and after 1 h of incubation to assess cellular metabolic activity. Metabolic changes were calculated from the difference between T0 and T1H values.

#### 2.6.4. Lactate and Ammonia Clearance

To evaluate the detoxification capacity of HepaRG-containing alginate beads, separate 6 h clearance assays were performed 3 days after thawing. For lactate clearance, beads were incubated in William’s E medium supplemented with 7 mM sodium L-lactate (cat. no. L7022; Sigma-Aldrich, St. Louis, MO, USA). For ammonia clearance, separate beads were incubated in a medium supplemented with a 2 mM solution of ammonium chloride (Sigma Aldrich, 213330). One-milliliter supernatant samples were collected at 0 and 6 h, and lactate and ammonia concentrations were measured using an Indiko clinical chemistry analyzer (hermo Fisher Scientific Oy, Vantaa, Finland). Clearance was calculated from the decrease in concentration between 0 and 6 h and normalized per hour and per 10^6^ cells. These assays were performed only for CRF conditions.

### 2.7. Statistical Analysis

All results were expressed as mean ± standard deviation (SD) unless otherwise specified. Statistical analyses were performed using GraphPad Prism software version 10.5.0 (GraphPad Software, San Diego, CA, USA). Statistical analyses were performed using the Kruskal–Wallis test followed by Dunn’s multiple-comparisons test. A *p*-value < 0.05 was considered statistically significant. An independent biological replicate was defined as a separate cell-culture, encapsulation, and cryopreservation experiment. Technical replicates within each biological experiment were averaged before statistical analysis. PrestoBlue included *n* = 4 biological replicates, whereas the other functional assays included *n* = 3 because of endpoint-specific sample availability. Each independently prepared encapsulation batch was considered one biological replicate, whereas measurements performed on aliquots derived from the same batch were considered technical replicates and were averaged before statistical analysis.

## 3. Results

### 3.1. Bead Morphology

**Figure 2:**
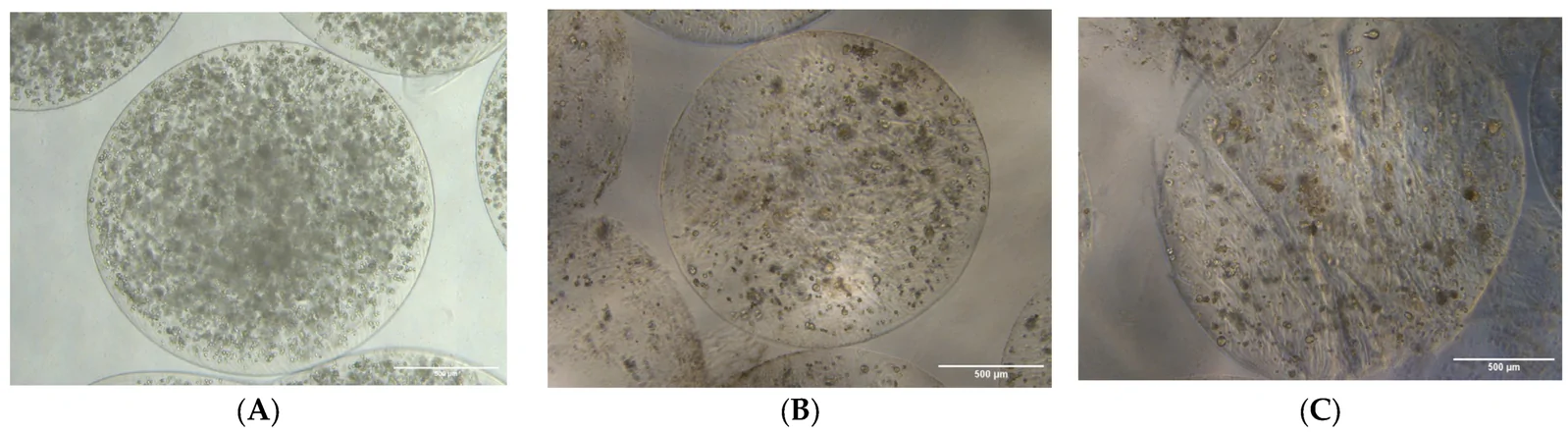
Bright-field images of HepaRG cells in 1.5% alginate beads: (**A**) unfrozen control at day 14, (**B**) 3 days after the CRF protocol, and (**C**) 3 days after passive freezing. Scale bar = 500 µm.

Prior to cryopreservation, samples were observed daily to ensure that the beads had a regular and uniform size (1.5 mm diameter) and no signs of alginate degradation or aggregation of the beads (samples with any of these particularities were discarded). The impact of the freezing strategy on the structural integrity of alginate-encapsulated HepaRG cells was evaluated after thawing. No apparent differences in bead morphology were observed between the unfrozen control (Figure 2A) and the controlled-rate freezing (CRF) condition (Figure 2B), with both maintaining a preserved spherical structure and intact alginate matrix. Conversely, conventional passive freezing led to noticeable morphological changes compared with the unfrozen control and CRF conditions, including surface irregularities and partial disruption of the alginate matrix (Figure 2C).

These alterations were consistently observed independently of the cryopreservation formulation, suggesting that the conventional freezing process adversely affected the physical stability of the alginate-based constructs.

### 3.2. Cell Viability

**Figure 3:**
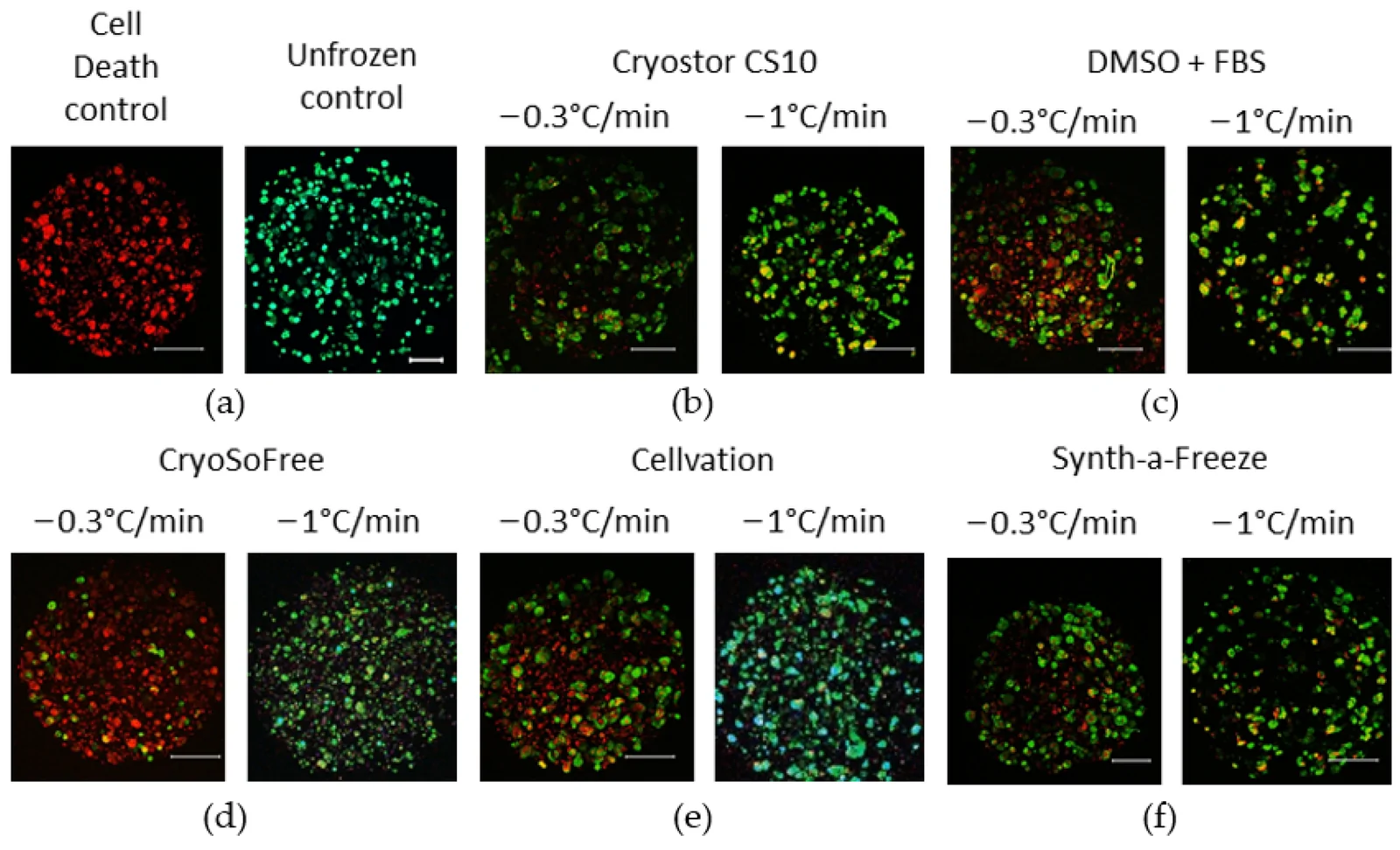
Qualitative Live/Dead imaging of alginate-encapsulated HepaRG cells 3 days after thawing under the indicated media and freezing protocols. (**a**) Cell-death control and unfrozen control; (**b**) CryoStor CS10; (**c**) DMSO + FBS; (**d**) CryoSoFree; (**e**) Cellvation; and (**f**) Synth-a-Freeze. On panels (**b**–**f**), the left and right images correspond to cooling rates of −0.3 °C/min and −1 °C/min, respectively. Live cells are shown in green (calcein-AM), live cells are green (calcein-AM), dead cells are red (ethidium homodimer-1), and nuclei are blue (Hoechst 33342). Scale bar = 200 µm.

Cell viability was assessed using a Live/Dead assay 72 h following cryopreservation across the different conditions, including a negative control for viability (Figure 3).

Live/Dead staining was used exclusively to provide a qualitative assessment of post-thaw membrane integrity across the tested cryopreservation media and freezing protocols. The cell-death and unfrozen controls are shown in Figure 3a. Three days after thawing, both live-associated green fluorescence and dead-associated red fluorescence were observed under both freezing protocols for CryoStor CS10 (Figure 3b), DMSO + FBS (Figure 3c), CryoSoFree (Figure 3d), Cellvation (Figure 3e), and Synth-a-Freeze (Figure 3f), indicating the coexistence of viable and non-viable cells in all post-thaw conditions. However, because the image acquisition parameters were not identical across conditions, fluorescence intensities and green-to-red signal ratios could not be directly compared or reliably quantified. Consequently, these images were not used to rank the cryopreservation conditions or to determine an interaction between the freezing protocol and the cryopreservation medium. Comparative conclusions regarding post-thaw recovery and functionality were therefore based on the quantitative assays presented below.

### 3.3. Activity of Cells

#### 3.3.1. Total Activity Measured by Presto BlueTM Assay

**Figure 4:**
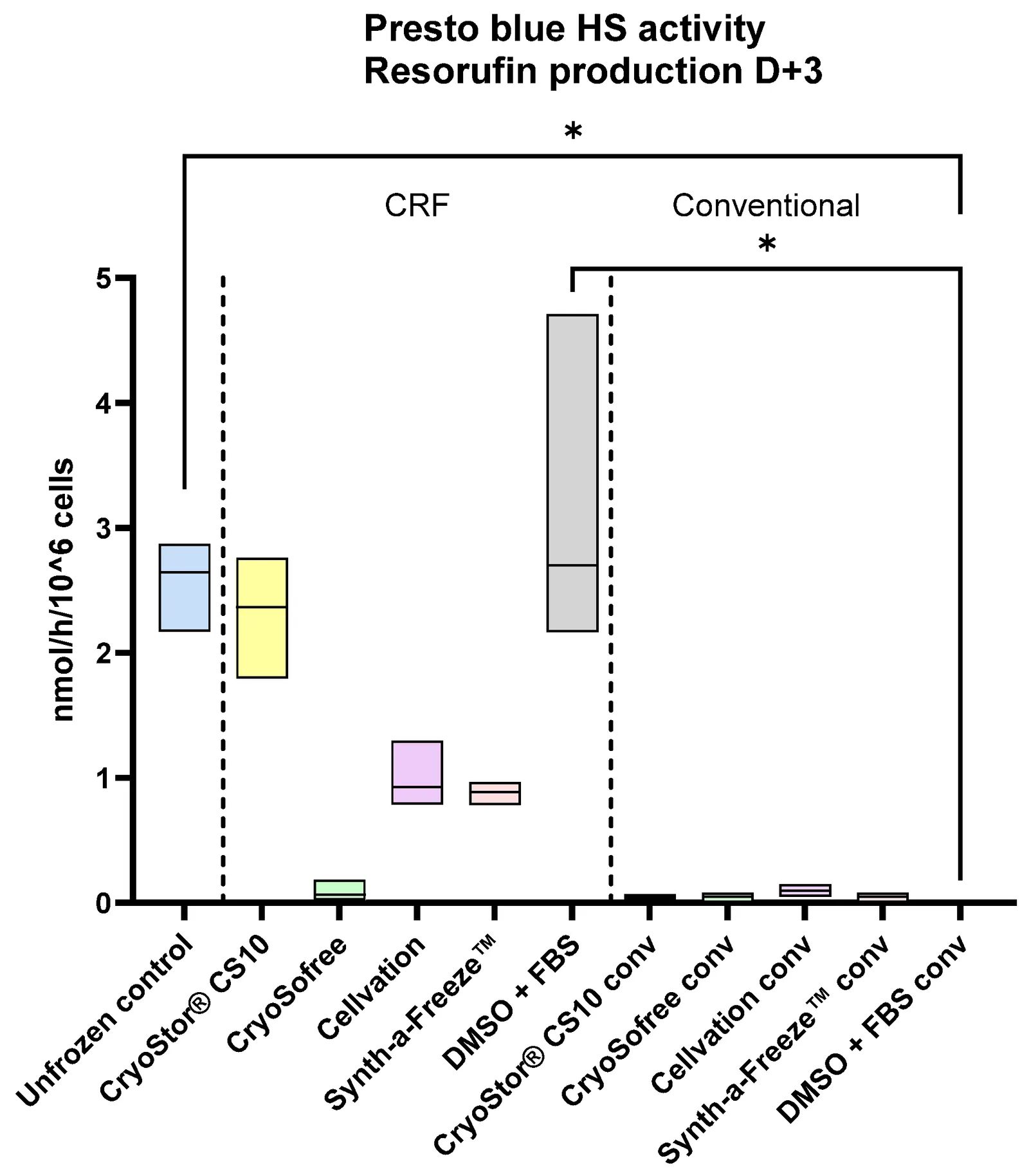
Metabolic activity measured with PrestoBlue HS and expressed as resorufin production per hour per 10^6^ cells. Kruskal–Wallis test followed by Dunn’s multiple-comparisons test (*n* = 4 independent biological replicates; * *p* < 0.05). Brackets indicate the compared groups.

To assess the impact of cryopreservation on mitochondrial metabolic activity, a PrestoBlue HS assay was performed 72 h post-thaw (D3) (Figure 4).

PrestoBlue HS analysis revealed that cryopreservation produced different mean metabolic activities across the tested protocol–medium combinations.

Following CRF, mean metabolic activity was 3.070 ± 1.1 nmol resorufin/h/10^6^ cells for the 90% FBS/10% DMSO formulation, 2.322 ± 0.45 for CryoStor^®^ CS10, 0.99 ± 0.24 for Cellvation, 0.88 ± 0.095 for Synth-a-Freeze™, and 0.08 ± 0.07 for CryoSoFree™. These means are described without implying pairwise significance beyond the comparisons indicated in Figure 4.

Following passive freezing, mean metabolic activity was 0.05 ± 0.02 nmol resorufin/h/10^6^ cells for CryoStor^®^ CS10, 0.04 ± 0.04 for CryoSoFree™, 0.10 ± 0.04 for Cellvation, 0.05 ± 0.04 for Synth-a-Freeze™, and 0.0008 ± 0.001 for the FBS/DMSO formulation. Conditions are not ranked solely from these mean values.

#### 3.3.2. Albumin Secretion Measurement by ELISA Assay

**Figure 5:**
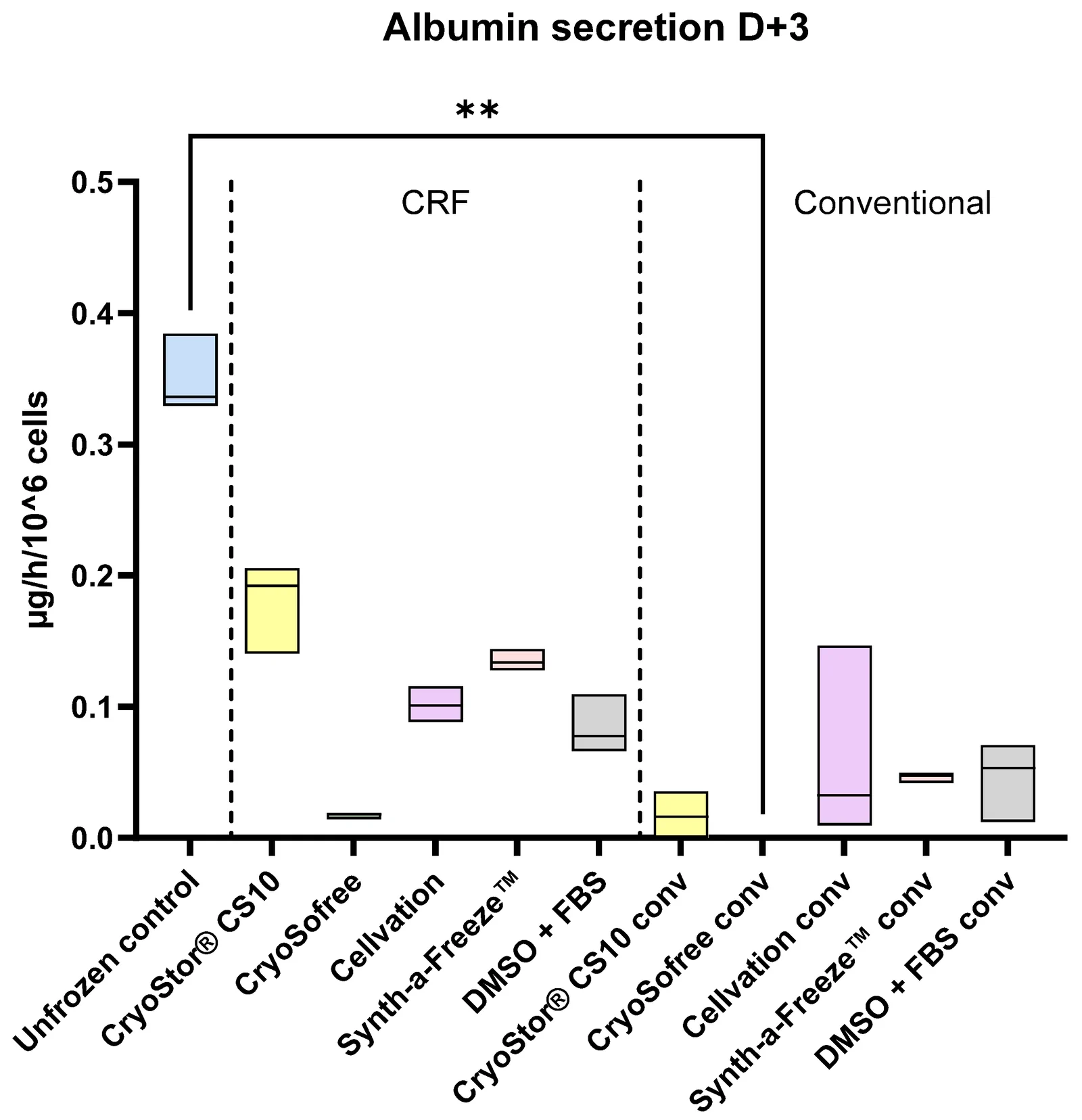
Albumin secretion rate per hour per 10^6^ cells. Kruskal–Wallis test followed by Dunn’s multiple-comparisons test (*n* = 3 independent biological replicates; ** *p* < 0.005). Brackets indicate the compared groups.

Albumin secretion, used as a marker of hepatocyte-specific synthetic function, was evaluated 3 days after thawing (Figure 5). Albumin secretion was reduced by cryopreservation compared to the unfrozen control.

Following CRF, albumin secretion remained detectable in all media. Mean secretion rates were 0.18 ± 0.03 µg/h/10^6^ cells for CryoStor^®^ CS10, 0.13 ± 0.008 for Synth-a-Freeze™, 0.10 ± 0.014 for Cellvation, 0.08 ± 0.02 for the 90% FBS/10% DMSO formulation, and 0.02 ± 0.002 for CryoSoFree™. These values should not be interpreted as a definitive ranking without the indicated pairwise comparisons.

Following passive freezing, mean albumin secretion rates were 0.017 ± 0.018 µg/h/10^6^ cells for CryoStor^®^ CS10, 0.000 for CryoSoFree™, 0.06 ± 0.06 for Cellvation, 0.05 ± 0.003 for Synth-a-Freeze™, and 0.045 ± 0.03 for the FBS/DMSO formulation.

#### 3.3.3. Phase I and II Detoxification Measurements

**Figure 6:**
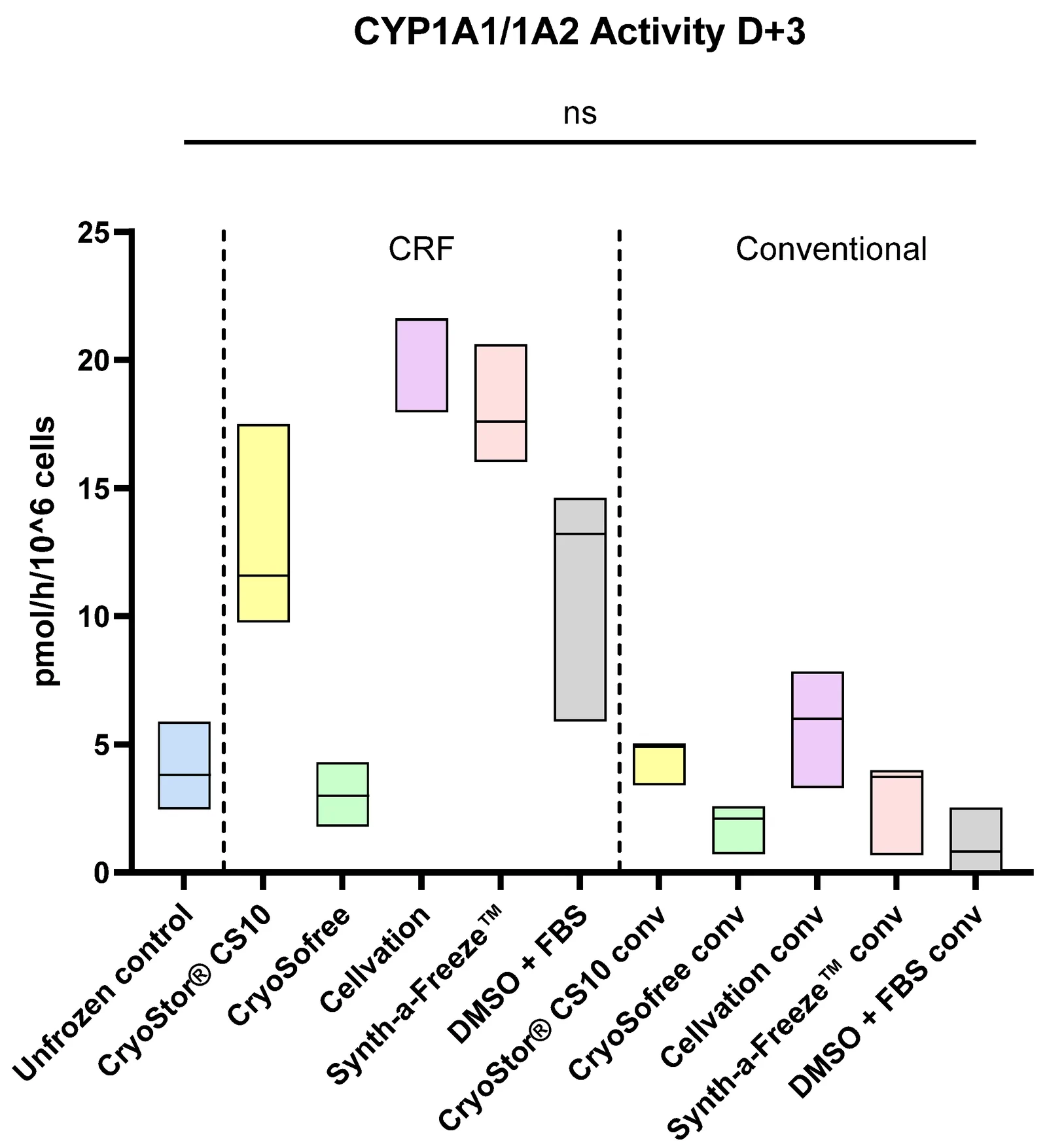
CYP1A1/1A2 activity measured using the EROD assay. Kruskal–Wallis test followed by Dunn’s multiple-comparisons test (*n* = 3 independent biological replicates; ns, *p* ≥ 0.05). Brackets indicate the compared groups; a spanning ns bar denotes a non-significant comparison.

The preservation of hepatic xenobiotic metabolism was assessed by measuring CYP1A1/1A2 activity as a marker of phase I biotransformation and UGT activity as a marker of phase II conjugation capacity, 3 days after thawing (Figure 6). Mean enzymatic activities varied across the tested protocol–medium combinations.

For CYP1A1/1A2 activity, the unfrozen-control mean was 4.05 ± 1.71 pmol/h/10^6^ cells. Under CRF, mean activities were 20.4 ± 2.1 for Cellvation, 18.07 ± 2.3 for Synth-a-Freeze™, 12.94 ± 4.03 for CryoStor^®^ CS10, 11.24 ± 4.69 for the FBS/DMSO formulation, and 3.03 ± 1.25 for CryoSoFree™. Only Cellvation was significantly higher than the unfrozen control (*p* = 0.0183); the other apparent elevations were not statistically supported. Under passive freezing, mean values ranged from 1.11 ± 1.3 to 5.78 ± 1.9 pmol/h/10^6^ cells. Thus, lower activity under passive freezing refers to the corresponding CRF mean and does not imply that every pairwise comparison was significant.

**Figure 7:**
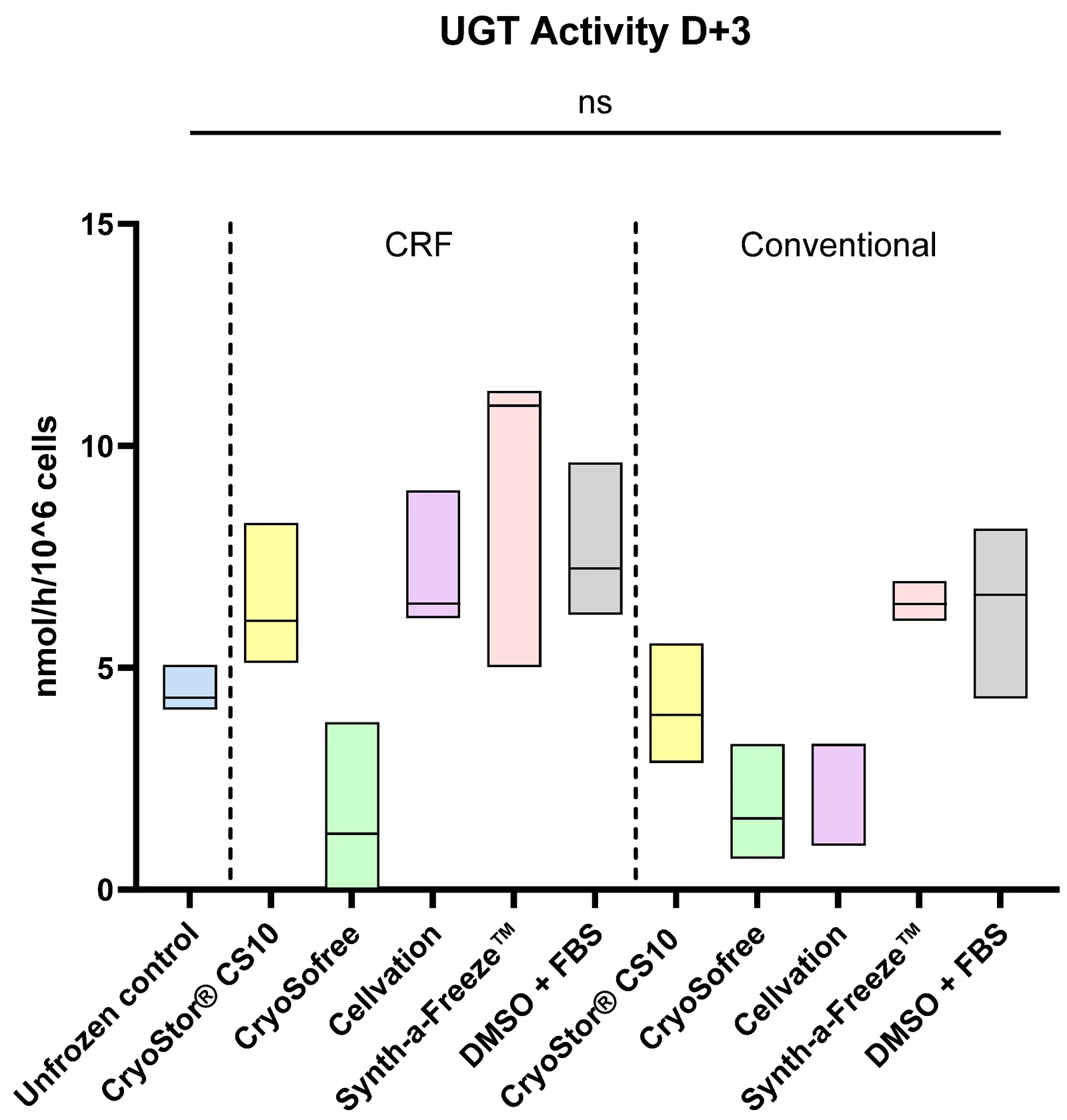
UGT1A1 activity measured using the 4-MU assay. Kruskal–Wallis test followed by Dunn’s multiple-comparisons test (*n* = 3 independent biological replicates; ns, *p* ≥ 0.05). Brackets indicate the compared groups; a spanning ns bar denotes a non-significant comparison.

For UGT1A1 activity, the unfrozen-control mean was 4.48 ± 0.52 nmol/h/10^6^ cells. Under CRF, mean activities were 6.48 ± 1.61 for CryoStor^®^ CS10, 1.68 ± 1.92 for CryoSoFree™, 7.19 ± 1.58 for Cellvation, 9.05 ± 3.5 for Synth-a-Freeze™, and 7.685 ± 1.76 for the FBS/DMSO formulation as shown in Figure 7. Under passive freezing, the corresponding means were 4.11 ± 1.35, 1.86 ± 1.31, 2.51 ± 1.32, 6.48 ± 0.45, and 6.36 ± 1.93 nmol/h/10^6^ cells. No reported UGT1A1 comparison was statistically significant; apparent elevations therefore do not demonstrate enhanced hepatic function (Figure 7).

Accordingly, the CYP1A1/1A2 and UGT1A1 values should be interpreted as endpoint-specific, product-level observations that require validation with additional biological replicates.

The limited replicate number and non-significant comparisons preclude concluding that the observed differences are biologically meaningful.

#### 3.3.4. Lactate and Ammonia Clearance

Because the tested CRF protocol generally produced higher short-term functional means and preserved bead morphology, lactate and ammonia clearance assays were performed only under CRF to compare media within that protocol. These endpoints were used as indicators of hepatocyte metabolic and detoxification activity after thawing.

**Figure 8:**
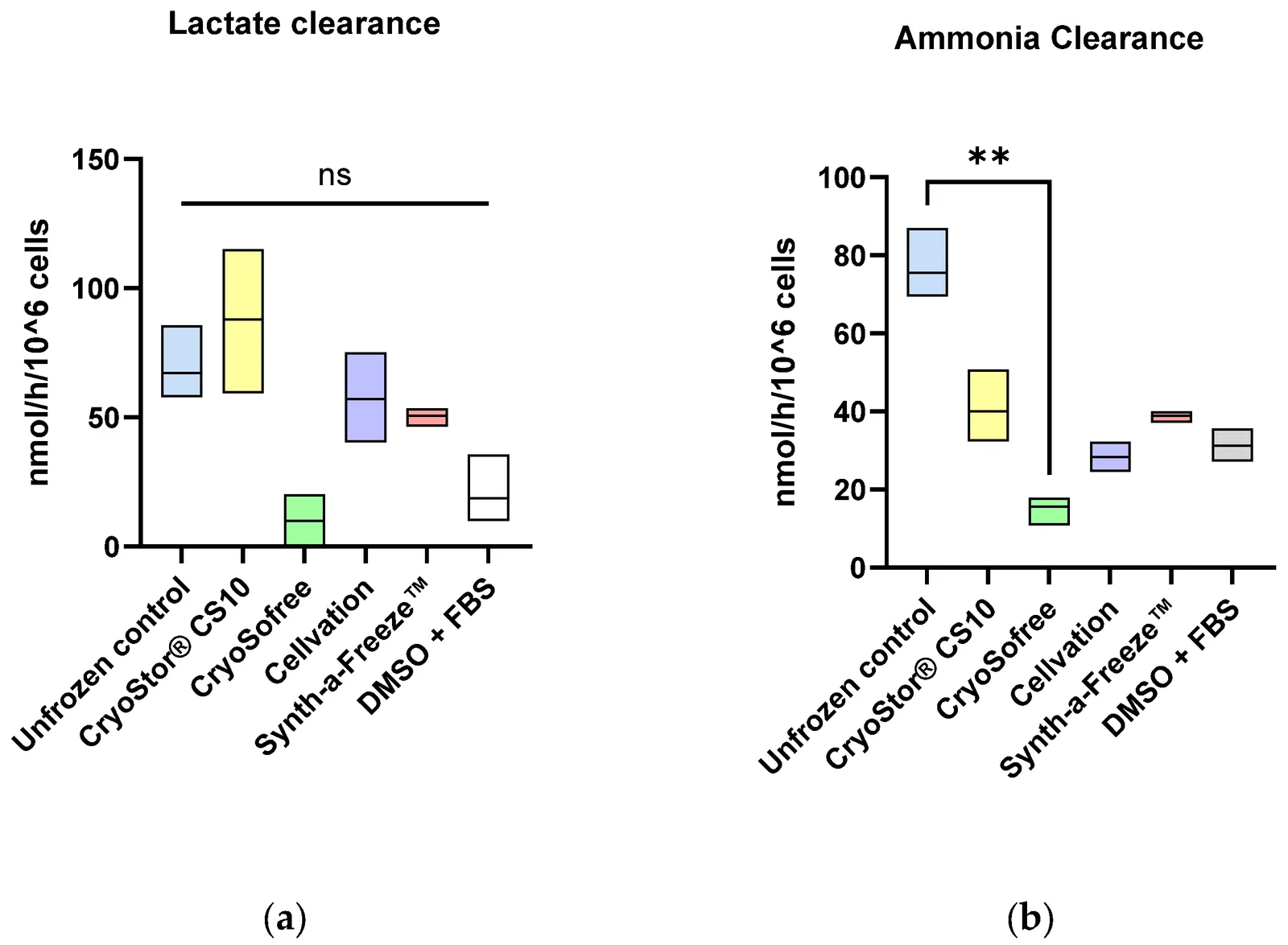
Clearance of (**a**) lactate and (**b**) ammonia by alginate-encapsulated HepaRG cells 3 days after thawing under CRF conditions. Kruskal–Wallis test followed by Dunn’s multiple-comparisons test (*n* = 3 independent biological replicates; ** *p* < 0.005; ns, *p* ≥ 0.05). Brackets indicate the compared groups; a spanning ns bar denotes a non-significant comparison.

Overall, cryopreserved encapsulated HepaRG cells demonstrated the ability to clear lactate (Figure 8a) and ammonia (Figure 8b) from the culture medium. The overall lactate comparison was not significant. For ammonia, CryoSoFree™ was significantly lower than the unfrozen control (*p* = 0.0029); no other reported adjusted pairwise comparison reached significance. The remaining differences are interpreted descriptively.

Lactate and ammonia clearance remained detectable 3 days after thawing under the tested CRF conditions.

Cryopreserved encapsulated HepaRG cells demonstrated the ability to clear lactate from the culture medium, with differences depending on the cryopreservation medium used. Overall, these results suggest that the capacity of encapsulated cells to detoxify xenobiotics appears to be strongly influenced by the cryopreservation conditions. Because these assays were not performed after passive freezing, they do not support a comparison between freezing protocols or conclusions about long-term detoxification capacity.

## 4. Discussion

In this study, we investigated two critical parameters governing the cryopreservation of alginate-encapsulated HepaRG cells: the freezing strategy (CRF vs. conventional) and the composition of the cryopreservation medium. The data provide a descriptive comparison of the tested protocol–medium combinations but do not isolate independent effects or a statistical interaction.

### 4.1. Protocol-Level Differences in Post-Thaw Structure and Function

The two complete freezing protocols were associated with different post-thaw outcomes. Under the tested CRF protocol, the alginate beads remained morphologically closer to unfrozen controls. Compared with the passive protocol, CRF appeared to better preserve bead integrity, whereas passive freezing was associated with surface irregularities and partial alginate disruption. Mean metabolic and hepatic functional readouts were also generally lower after passive freezing. These observations are consistent with previous studies [35,44,45,46], which describe benefits of controlled freezing profiles in encapsulated systems. However, the protocols differed not only in nominal cooling rate (−0.3 versus approximately −1 °C/min) but also in the −10 °C hold and the −80 °C transfer and storage sequence. The present data therefore cannot demonstrate that −0.3 °C/min is intrinsically superior to −1 °C/min; a design varying one process parameter at a time would be required.

### 4.2. Cryopreservation Negatively Impacts Cellular Activities

Because the tested CRF protocol generally produced higher short-term functional means, we investigated the effects of the commercially available xeno-free media on the viability and functionality of cryopreserved alginate-encapsulated HepaRG cells, with a particular focus on their metabolic and hepatic-specific activities three days post-thaw. As expected, a decrease in metabolic activity was observed at day 3 post-thaw, reflecting delayed cryoinjury commonly reported in cryopreserved cell systems [47]. This phenomenon is typically associated with osmotic imbalance, membrane damage, and mitochondrial dysfunction occurring after thawing, which can impair cell recovery despite initial survival [13,48,49,50].

Although cryopreserved cells showed evidence of delayed cryoinjury compared with unfrozen controls, measurable albumin secretion, xenobiotic metabolism, and ammonia and lactate clearance remained detectable at the single 3-day time point. Recovery patterns differed by endpoint, and the present comparisons do not establish a formulation that is statistically superior across all assays.

### 4.3. Viability

Live/Dead staining confirmed that viable cells remained detectable after thawing in all cryopreservation conditions, although the simultaneous presence of green and red fluorescence indicated incomplete preservation compared with the unfrozen control. Qualitatively, the conventional cooling protocol at −1 °C/min appeared to produce a stronger green signal than controlled-rate freezing at −0.3 °C/min for several cryopreservation media. However, this observation did not fully correlate with the metabolic assays, which showed a more favorable overall recovery following controlled-rate freezing. This apparent divergence can be explained by the fact that Live/Dead staining primarily evaluates membrane integrity at a specific time point, whereas metabolic and hepatic functional assays assess the capacity of the cells to recover and maintain their activity after thawing. Cells with intact membranes may therefore still exhibit sublethal cryoinjury, mitochondrial dysfunction or impaired liver-specific functions. Moreover, fluorescence intensity may be influenced by differences in cell density, staining penetration through the alginate matrix, image acquisition and field selection. Consequently, these images provide qualitative evidence of post-thaw cell survival but are insufficient to rank the cryopreservation conditions without fluorescence quantification. When considered together with the functional results, they highlight the importance of using a multiparametric evaluation combining viability, metabolic activity and liver-specific functions to identify the most suitable cryopreservation protocol.

### 4.4. Mitochondrial Activity and Albumin Secretion

The highest PrestoBlue mean signals under CRF were observed with the FBS/DMSO formulation and CryoStor^®^ CS10. However, Synth-a-Freeze™ also contains 10% DMSO and produced a lower mean; the present data therefore do not support a general advantage of DMSO-containing media. DMSO can influence mitochondrial and hepatic biology independently of cryoprotection [51]. In primary hepatocytes, 1% DMSO has been reported to increase mitochondrial dehydrogenase activity and albumin secretion [52].

Albumin secretion also varied by product: CryoStor^®^ CS10 had the highest CRF mean, whereas the 90% FBS/10% DMSO formulation had a lower mean despite the same declared DMSO concentration. DMSO can also modulate hepatocyte differentiation status [53]. The 90% FBS/10% DMSO formulation showed significantly lower albumin secretion than the unfrozen control (*p* = 0.0466). These results should be interpreted as product-specific rather than as a class effect of DMSO [54].

### 4.5. Xenobiotic Metabolism

Regarding phase I and II biotransformation, the mean response profiles differed among products and endpoints, but most comparisons were not significant. In the EROD assay, several CRF means exceeded the unfrozen-control mean. These increases may reflect transient post-thaw regulation, assay normalization, or formulation-specific effects rather than enhanced function. Previous studies show that DMSO can regulate CYP expression through transcriptional and epigenetic mechanisms [55]. However, Cellvation, which is DMSO-free, was the only medium with CYP1A1/1A2 activity significantly higher than the unfrozen control (*p* = 0.0183). DMSO alone therefore cannot explain the CYP pattern, and the remaining elevations should be treated as non-significant product-specific observations.

### 4.6. Ammonia and Lactate Removal

Ammonia and lactate clearance were assessed only after CRF, so these data cannot compare freezing protocols. CryoStor^®^ CS10 had the highest mean lactate clearance, but the overall lactate comparison was not significant. For ammonia, CryoSoFree™ was significantly lower than the unfrozen control (*p* = 0.0029); no other reported pairwise comparison was significant. These short-term measurements show detectable activity but do not establish long-term function or suitability for a bioartificial liver.

Xeno-free formulations may improve standardization and biosafety, but xeno-free status alone did not predict post-thaw recovery in this study. Medium selection should therefore be based on product-specific evidence for the intended cell type and endpoint.

While the presence or absence of DMSO may partly explain some of the metabolic differences observed between cryopreservation conditions, it is unlikely to represent the sole determinant of post-thaw hepatocyte functionality. Indeed, if DMSO alone were responsible for the preservation of hepatic metabolism, a consistent functional hierarchy would be expected across all assays, which was not observed in the present study. Instead, distinct cryopreservation media displayed variable performances depending on the metabolic endpoint considered, suggesting the involvement of additional factors influencing post-thaw recovery. Several parameters may contribute to these differences, including osmotic properties, viscosity, CPA permeability, antioxidant components, membrane-stabilizing agents, or other proprietary additives capable of modulating cellular stress responses and mitochondrial recovery. Furthermore, although commercial cryopreservation media are generally described according to broad characteristics such as DMSO content and serum-free formulation, their complete compositions remain largely undisclosed. Consequently, unidentified constituents present within these formulations may differentially influence hepatocyte metabolism, biotransformation activity, and post-thaw functional restoration.

These observations support product-specific interpretation and do not permit mechanistic attribution to DMSO alone.

Because media with the same declared DMSO content produced different results and complete commercial compositions were not disclosed, no general conclusion can be drawn for DMSO-containing versus DMSO-free media.

For studies focusing on xenobiotic metabolism, preservation of the relevant phase I and II activities should be evaluated directly; the present data do not establish a general advantage of DMSO-free media [56,57].

When DMSO-free conditions are required, alternative cryopreservation solutions must be considered because of the known cytotoxicity and potential adverse biological effects associated with DMSO exposure. In this regard, Cellvation Cryopreservation Medium is a candidate for further evaluation in DMSO-free applications. Its high mean CYP1A1/1A2 activity should be confirmed using quantitative viability, additional biological replicates, and longer post-thaw follow-up before the product is considered a superior alternative.

### 4.7. Study Limitations

This study is limited by the small number of independent biological replicates (*n* = 3 for most endpoints and *n* = 4 for PrestoBlue), the single short-term assessment at 3 days post-thaw, and the qualitative Live/Dead analysis. Moreover, the complete compositions of the commercial formulations were proprietary and may include undisclosed bioactive components.

## 5. Conclusions

The tested CRF protocol was associated with better apparent bead integrity and generally higher short-term functional means than passive freezing. Because the protocols differed in cooling rate, the −10 °C hold, and the −80 °C transfer and storage sequence, these data cannot attribute the difference to the −0.3 °C/min rate alone. The current endpoint-specific analyses also do not separate the independent effects of protocol and medium.

Cryopreserved alginate-encapsulated HepaRG cells retained measurable albumin secretion, xenobiotic metabolism, and ammonia and lactate clearance at 3 days post-thaw (Appendix A). No medium was consistently optimal across all endpoints. CryoStor^®^ CS10 produced relatively high mean values for several CRF readouts, whereas DMSO-free Cellvation produced the only CYP1A1/1A2 value significantly above the unfrozen control; both observations require confirmation with greater statistical power. These findings support the short-term feasibility of the tested CRF protocol for this encapsulated HepaRG model but do not establish suitability for drug testing, toxicology, cell transplantation, or bioartificial liver use.

## Figures and Tables

**Table 1:** Cryopreservation media and manufacturer-declared DMSO and xeno-free status; complete commercial compositions are proprietary.

Cryopreservation Medium	Supplier	DMSO	Xeno-Free
Cryostor CS10	Stemcell^TM^ Technologies Vancouver, BC, Canada	10%	YES
CryoSoFree	Sigma Aldrich St. Louis, MO, USA	DMSO-free (manufacturer-declared)	YES
Cellvation Cryopres Medium	MP Biomedicals Solon, OH, USA	DMSO-free (manufacturer-declared)	YES
Synth-a-Freeze^TM^	Thermofischer Scientific Waltham, MA, USA	10%	YES
10% DMSO + 90% FBS	In House Medium	10%	NO

## Data Availability

The data presented in this study are available on request from the corresponding author.

## Abbreviations

The following abbreviations are used in this manuscript:

3D	Three-dimensional
CPA	Cryoprotective agent
CRF	Controlled-rate freezing
CYP1A	Cytochrome P450 family 1 subfamily A
DMSO	Dimethyl sulfoxide
EDTA	Ethylenediaminetetraacetic acid
ELISA	Enzyme-linked immunosorbent assay
EROD	7-Ethoxyresorufin-O-deethylase
FBS	Fetal bovine serum
PBS	Phosphate-buffered saline

## Appendix A

Detailed data from the metabolic assays performed on encapsulated hepatocytes.

**Table A1:** Summary of the different metabolic activities of the encapsulated HepaRG depending on the cryopreservation media and method.

		CRF (−0.3 °C/min)					Conventional Freezing (−1 °C/min)				
CPA	Unfrozen Control	Cryostor^®^ CS10	CryoSoFree	Cellvation	Synth-A-Freeze TM	10% DMSO + 90% FBS	Cryostor^®^ CS10	CryoSoFree	Cellvation	Synth-A-Freeze TM	10% DMSO + 90% FBS
CYP1A1/1A2 pmol/h/10^6^ cells	4.048 ± 1.709	12.94 ± 4.033	3.029 ± 1.247	20.39 ± 2.099	18.07 ± 2.333	11.24 ± 4.685	4.440 ± 0.8988	1.794 ± 0.9703	5.781 ± 1.898	2.791 ± 1.834	1.111 ± 1.288
UGT nmol/h/10^6^ cells	4.480 ± 0.5212	6.477 ± 1.613	1.678 ± 1.922	7.186 ± 1.579	9.052 ± 3.504	7.685 ± 1.760	4.114 ± 1.350	1.863 ± 1.310	2.511 ± 1.318	6.480 ± 0.4487	6.361 ± 1.927
Albumin secretion µg/h/10^6^ cells	0.3501 ± 0.02984	0.1794 ± 0.03428	0.01713 ± 0.002398	0.1016 ± 0.01357	0.1351 ± 0.008047	0.08442 ± 0.02237	0.01719 ± 0.01766	0.000 ± 0.000	0.05525 ± 0.06333	0.04661 ± 0.003198	0.04550 ± 0.02990
Presto Blue (resorufin production) nmol/h/10^6^ cells	2.584 ± 0.3263	2.322 ± 0.4521	0.08443 ± 0.07083	0.9852 ± 0.2360	0.8814 ± 0.09456	3.070 ± 1.127	0.05146 ± 0.02001	0.04463 ± 0.04100	0.09932 ± 0.04109	0.04463 ± 0.04100	0.0008047 ± 0.001394
Lactate clearance nmol/h/10^6^ cells	67.13 ± 15.98	87.94 ± 27.81	9.965 ± 10.14	57.05 ± 17.46	50.70 ± 3.725	18.64 ± 14.74	N/D	N/D	N/D	N/D	N/D
Ammonia clearance nmol/h/10^6^ cells	75.50 ± 10.02	40.01 ± 9.534	15.59 ± 4.111	28.39 ± 3.847	38.96 ± 1.604	31.18 ± 4.212	N/D	N/D	N/D	N/D	N/D
